# Allogeneic Hematopoietic Stem Cell Transplantation in Patients with Prolymphocytic Leukemia

**DOI:** 10.3390/jcm14082816

**Published:** 2025-04-19

**Authors:** Christina Groiss, Stefanie Kreissl, Irene Strassl, Olga Saini, Dagmar Wipplinger, Robert Milanov, Emine Kaynak, Petra Hasengruber, Christoph Aichinger, Stefanie Nocker, Thomas Bauer, Veronika Buxhofer-Ausch, Sigrid Machherndl-Spandl, Michaela Binder, Alexander Nikoloudis, Michael Girschikofsky, Andreas Petzer, Ansgar Weltermann, Johannes Clausen

**Affiliations:** 1 Division of Hematology with Stem Cell Transplantation, Hemostaseology and Medical Oncology, Department of Internal Medicine I, Ordensklinikum Linz Elisabethinen, Fadingerstrasse 1, 4020 Linz, Austria; 2 Medical Faculty, Johannes Kepler University Linz, Altenberger Strasse 69, 4040 Linz, Austria

**Keywords:** allogeneic hematopoietic stem cell transplantation, T-prolymphocytic leukemia, B-prolymphocytic leukemia

## Abstract

**Background:** T-prolymphocytic leukemia (T-PLL) is a rare lymphoid neoplasm with particularly poor prognosis. Although it is no longer recognized as a distinct entity by the World Health Organization (WHO), B-prolymphocytic leukemia (B-PLL) comprises conditions with unfavorable outcomes. Both diseases most frequently affect patients in the seventh decade of their lives. Allogeneic hematopoietic stem cell transplantation (alloHSCT) significantly improves outcomes for selected PLL cases, as shown by several, mostly retrospective, analyses. **Methods:** In this article, we provide a review of existing PLL analyses, followed by a summary of cases treated at our center. We describe outcomes of six T-PLL and three B-PLL cases receiving alloHSCT at our institution between 2015 and 2022. **Results:** Despite a post-transplant 4-year cumulative relapse incidence of 61% in our T-PLL series, the median OS was 78 months, because relapse therapy was remarkably successful. All B-PLL patients are alive and relapse-free, with a median follow-up of 54 (range of 11–74) months. A poor pre-transplant Karnofsky performance status (KPS) (≤ 80%) and an HCT comorbidity index (HCT-CI) of ≥3 were significantly associated with post-transplant mortality. **Conclusions:** The comparatively favorable outcomes in our case series underline the increasing value of alloHSCT in PLL in the current era, as it offers a prospect of cure in selected patients with otherwise very poor prognosis.

## 1. Introduction

### 1.1. T-PLL

#### 1.1.1. Definition/Discovery

According to the fifth edition of the World Health Organization (WHO) classification of lymphoid neoplasms, T-prolymphocytic leukemia (T-PLL) is one of six types of mature natural killer (NK)-cell and T-cell neoplasms, characterized by divergent individual clinical development [1] and progressive marked peripheral lymphocytosis [2]. Catovsky et al. first described this lymphoproliferative disorder in 1973, which significantly differs from cases of chronic lymphocytic leukemia (CLL) regarding pathological, clinical and laboratory features [3]. Matutes et al. further reported distinct immunophenotypic features (the most common phenotype of T-prolymphocytes being CD4+/CD8−) and cytogenetic changes with involvement of chromosome 14 [2]. Considering the maturity and post-thymic origin of the cells, the term “prolymphocytes” appears to be a misnomer [3,4].

#### 1.1.2. Epidemiology

Despite generally being a rare disease with an overall incidence of 0.6/Mio./year [4], T-PLL still represents the most frequently diagnosed mature T-cell leukemia with 2/Mio./year in Western countries [5], constituting one-third of mature T-cell-leukemias [2]. However, T-PLL accounts for only 2% of all lymphoid leukemias in adults >30 years [6,7]. The median age at time of diagnosis is about 65 years (range, 33–91 years) [2,8,9]. T-PLL is slightly more common in male patients, with a reported male/female ratio of 1.33 [2,8]. Although most cases occur sporadically, there are certain genetic conditions with an increased risk of developing T-PLL, namely ataxia telangiectasia and Nijmegen breakage syndrome [2,4,6,7,10].

#### 1.1.3. Diagnosis

For establishing the diagnosis of T-PLL, evaluation of peripheral blood smear, immunophenotype and genetic features of the malignant T-lymphocytes need to be performed. Furthermore, T-cell receptor rearrangement (by polymerase chain reaction (PCR) for T-cell receptor beta and/or gamma chains, or by flow cytometry) and testing for human T-lymphotropic virus (HTLV) in endemic countries are mandatory at initial diagnosis, whereas bone marrow aspirate/biopsy is only required to assess response to treatment [4,6,8,11].

Staber et al. published new consensus criteria in 2019, unifying diagnostic requirements: all three major criteria (i.e., >5 × 10^9^/L cells of T-PLL phenotype in peripheral blood or bone marrow; T-cell-clonality; abnormality of 14q32 or Xq28 OR expression of TCL1A/B or MTCP1) and at least one of four minor criteria (abnormalities involving chromosome 11 or chromosome 8; abnormalities in chromosome 5, 12, 13, 22 or complex karyotype; involvement of T-PLL specific site) need to be met [4].

#### 1.1.4. Immunophenotyping

The mature, post-thymic phenotype of T-PLL-cells—although it appears highly heterogenous—can be verified by immunophenotyping. With about two-thirds, CD4+CD8- appears to be the most common pattern in T-PLL cells, followed by co-expression of CD4 and CD8 in about one-third of cases [9]. Cases of CD4-CD8+ and CD4-CD8- have also been reported [12]. The mandatory markers are CD2, CD3, CD5. Moreover, CD7 and CD52 are typically strongly expressed [2,6,7,8,9,11,12,13].

#### 1.1.5. Cytogenetics

Recognizing alterations on chromosome 14 and X—with consecutive overexpression of the proto-oncogenes TCL1 and MTCP1, respectively—as the hallmark of the disease is crucial for understanding the pathophysiology of T-PLL [12]. The overexpression of these proto-oncogenes is caused by rearrangement between those and the T-cell receptor (TCR) genes on chromosome 14 [6]. Notably, TCL1 overexpression alone is not sufficient for the development of T-PLL; secondary events appear to be essential [9]. About 70% of T-PLL cases appear to have a complex karyotype, especially ≥ 5 aberrations, which are associated with worse prognosis [14].

#### 1.1.6. Molecular Genetics

On the molecular level, several genetic mutations contributing to T-PLL pathogenesis and evolution have been discovered so far. These include gain of function mutations e.g., concerning JAK1/3, STAT5B and IL2RG, as well as loss of function mutations regarding deoxyribonucleic acid (DNA) repair and epigenetic regulation (e.g., EZH2) [7,11,12,15].

#### 1.1.7. Clinical Manifestations

Two phases in the course of the disease can be discriminated: in up to one-third of the cases, T-PLL is diagnosed in an indolent/inactive phase, which might last up to several years [6,7,11,15,16]. However, all patients will undergo progression into active disease, eventually requiring treatment [2,4,16]. The conversion into active, symptomatic disease derives from increasing tumor burden—no phenotypic or other measurable changes in T-PLL cells could be detected [4,9].

Typically, a brief phase (approx. 2 months) of constitutional symptoms and splenomegaly (present in 80% of the patients) precedes the diagnosis of T-PLL; in 50%, non-bulky lymphadenopathy is evident [6,9,13]. At time of diagnosis, most patients will present with white blood cell (WBC) count > 100 × 10^9^/L with >90% lymphocytes; 30% of patients will also show anemia and thrombocytopenia. Slightly elevated lactate dehydrogenase (LDH) levels are also common [8,16]. Further characteristic clinical signs include skin manifestations (e.g., maculopapular rash, effusions or nodules), which occur in 25% of the cases; pleuro–peritoneal effusions and, with 10% less frequently, involvement of the central nervous system (CNS) [9,13,16]. Dearden et al. described periorbital and/or conjunctival edema as a distinctive clinical feature of T-PLL [16].

#### 1.1.8. Prognosis

With a median overall survival (OS) of approx. 20 months [7,9] and a 5-year survival rate of 21% [17], the general prognosis of T-PLL appears rather poor. Prognostic factors associated with impaired OS include complex karyotype [14], LDH levels > 1668 U/L and beta-2-microglobulin (B2M) > 8 mg/L [18]. Moreover, in a retrospective analysis of 86 T-PLL cases, older age at presentation (>62 years), high expression of the TCL-1 protein, higher peak WBC count, and a lymphocyte doubling time < 8.5 months correlated with worse outcomes [17].

According to Dearden et al., response to alemtuzumab therapy is a crucial predictor of outcome [16]. In the setting of relapsed disease, prognosis is even worse, with reported OS rates of 6–9 months [4] and a 5-year OS < 5% [5].

#### 1.1.9. Treatment

Treatment indication is determined by the diagnosis of “active disease”; thus, T-PLL-related symptoms need to be present. These are constitutional symptoms (fatigue or B-symptoms); bone marrow failure indicated by anemia or thrombocytopenia; rapidly enlarging lymph nodes/spleen/liver; increasing lymphocytosis or extranodal involvement. In the rare cases of “inactive T-PLL”, monthly diagnostic workups should be performed to assess disease progression. There is no evidence that treatment initiation in this phase would be beneficial [4].

Previously, the treatment of choice was polychemotherapy containing alkylating agents (e.g., cyclophosphamide, doxorubicin, vincristine, prednisone—CHOP). If achieved at all, responses were incomplete and of short duration, with a median survival of seven months [2,8]. The treatment of choice nowadays is alemtuzumab (CAMPATH-1H), a fully humanized IgG1 kappa monoclonal antibody against CD52—an antigen strongly expressed on the surface of practically all T-PLL cells [7,13]. Alemtuzumab should be administered intravenously, as subcutaneous application has been associated with significant impaired overall response (ORR) rates of 33% (versus 91% when given intravenously) [19]. As a first-line therapy, alemtuzumab induces overall response rates > 90% [4,7] with reported progression-free survival (PFS) of one year [16].

While routine CNS prophylaxis is not recommended, patients with CNS involvement should receive intrathecal triple therapy (methotrexate, cytarabine, hydrocortisone) or high-dose systemic methotrexate in addition to treatment with alemtuzumab [16].

The combination of fludarabine, mitoxantrone and cyclophosphamide (FMC) induced ORR rates of 68% in a prospective multicenter phase 2 trial performed by Hopfinger et al. [20]. However, their benefit over alemtuzumab monotherapy could not be shown; therefore, FMC should only be administered in cases of severe intolerance of alemtuzumab or in refractory/relapsed (r/r) situations [15,20]. Furthermore, nucleoside analogs like pentostatin might be added to alemtuzumab if response to monotherapy is insufficient [6,15]. Ravandi et al. could prove the effectiveness of a combined therapy of alemtuzumab and pentostatin with ORR of 69% and an OS of 10.2 months [21]. The alkylating agent bendamustin could induce ORR of 53%, with OS rates of 8.7 months in a retrospective study performed by Herbaux et al. [22]. Both agents did not lead to superior outcomes compared to alemtuzumab monotherapy, and are therefore only recommended in salvage situations, i.e., alemtuzumab r/r cases [21,22].

For patients ineligible for allogeneic hematopoietic stem cell transplantation (alloHSCT), autologous hematopoietic stem cell transplantation (autoHSCT) after high-dose chemotherapy offers a reasonable alternative, with significantly lower rates of treatment-related mortality (TRM) [16], still leading to prolonged OS and PFS compared to the non-transplant-setting. However, significantly higher relapse rates occur compared to alloHSCT, which make long-term-survival very unlikely [23]. Therefore, alloHSCT should be the consolidation treatment of choice, whenever the patient’s performance status allows it and a suitable donor is available [7].

#### 1.1.10. Allogeneic Hematopoietic Stem Cell Transplantation

Three retrospective analyses were published until 2012, all describing alloHSCT in T-PLL patients with a median age of approximately 51 years. Two of the analyses had similar 3-year OS rates (Krishnan et al. 38%; Guillaume et al. 36%), and Jedrezecjak et al. reported lower 3-year OS rates of 21% [23,24,25].

In the allogeneic cohort of Krishnan et al.’s study published in 2010, the rates of the 13 patients receiving alloHSCT in CR or partial remission (PR) was remarkably high, with 69% and 31%, respectively [23]. Relapse rates of 31% and TRM rates of 31% were reported, the latter only in patients who underwent myeloablative conditioning.

Guillaume et al. described alloHSCT in 27 T-PLL patients of whom 52% were in CR and 37% in PR at time of transplantation [25]. They observed 3-year-relapse rates of 47% (half of the relapses occurring within the first year) and TRM-rates of 31%. In 59% of the cases, reduced intensity conditioning (RIC) was applied and total body irradiation (TBI) was part of 56% of the conditioning regimens.

In 2012, Jedrzejcak et al. analyzed outcomes of 41 T-PLL-alloHSCT patients and observed identical 3-year relapse and NRM rates of both 41% [24]. A total of 27% of the patients were in CR at time of transplantation, and 29% were in PR. Via multivariate analysis, the use of TBI and a shorter interval (<12 months) between diagnosis and alloHSCT were associated with longer PFS.

Kalaycio et al. described PFS-rates of 5.1 months for T-PLL patients receiving alloHSCT [26]. A total of 75% of the 47 patients analyzed (importantly, only 21 of those suffered from T-PLL while 11 patients had B-PLL, and in 15 cases, the type of PLL was unknown) had a Karnofsky performance status (KPS) >80%, and 36% were in complete remission (CR) at time of transplant. The median time from diagnosis to transplant was 11 months.

In contrast to the studies discussed above, the single-center retrospective analysis of 119 T-PLL patients published by Jain et al. in 2017 showed no significant difference in OS and PFS with or without alloHSCT carried out in first CR [18]. Despite a longer follow-up period of 27 years, the lack of benefit cannot yet be fully explained.

In Table 1 and Table 2, patient and transplant characteristics and outcomes of the studies mentioned above are summarized.

To prevent engraftment failures caused by in vivo T-cell depletion, a time interval of 3 months between last administration of alemtuzumab and alloHSCT is recommended [23,28]. Nevertheless, Jedrzejczak et al. could not find an adverse effect of persisting alemtuzumab serum levels on disease control [27].

#### 1.1.11. Response Assessment

In the consensus guidelines published in 2019, Staber et al. unified response criteria to aid comparison of clinical studies [4]. A selection of clinical features (size reduction in lymph nodes, spleen, absence of constitutional symptoms and other clinical manifestations) and decrease in circulating lymphocyte count as well as hematological regeneration measured by hemoglobin, neutrophil and platelet count are assessed. Notably, confirmation of the response via bone marrow examination (aspirate and biopsy) is required.

#### 1.1.12. New Approaches

Considering the overall dismal prognosis of T-PLL cases, especially in alemtuzumab r/r situations, there is urgent need for new therapeutic approaches [15]. This is particularly important for preemptive treatment to target residual T-PLL cells and thus prevent relapse after alloHSCT [27], which usually occurs within three years with highest incidence in year one, and is associated with exceptionally poor outcomes [6].

Recent ex vivo and in vivo studies tested the activity and effectiveness of drugs, including histone deacetylase inhibitors (anti-HDAC), phosphoinositide-3 kinase (PI3K) inhibitors, cycline-dependent kinase (CDK9)-inhibitors, B-cell lymphoma (BCL)-inhibitors, Janus-activated kinase-signal transducer and activator of transcription factor (JAK/STAT) pathway inhibitors, poly-ADP-ribose polymerase (PARP) inhibitors and p53 activators [7,27].

The B-cell lymphoma 2 (BCL-2)–selective small-molecule inhibitor venetoclax is of particular interest, as Boidol et al. could show strongest ex vivo response out of 106 different agents after applying it to single-cell suspensions of 86 patient samples [29]. Subsequently, venetoclax was administered to two r/r T-PLL patients who thereby both achieved PR. Response to venetoclax correlated with BCL-2 expression levels, but not with BCL extra-long (BCL-XL) or myeloid cell leukemia 1 (MCL-1) expression levels. MCL-1 expression levels even inversely correlated with those of BCL-2. However, in order to prevent the development of venetoclax resistance via BCL-2 or BCL-XL induction, more studies on combination therapies with other agents are crucial [29,30]. For example, CDK9 inhibitors, which cause MCL-1 downregulation and p53 activation, constitute a synergistic strategy [31].

By high-throughput ex vivo drug-testing, Andersson et al. discovered that, despite the high occurrence of JAK-STAT pathway mutations in T-PLL-cases, responses to JAK-STAT inhibitors did not correlate with the same [31]. Nevertheless, JAK-STAT inhibitors represent a potential efficient, usually well-tolerated agent [32]. The JAK1/2 inhibitor ruxolitinib or anti-HDAC belinostat were shown to increase sensitivity to venetoclax. First, in vivo experiences performed by Herbaux et al. showed promising results [33]. Further epigenetic modulators, such as vorinostat or romidepsin, might also induce expression of targetable markers such as CD30 in T-PLL cells, which consequently makes treatment with brentuximab vedotin feasible [34].

CAR-NK (chimeric antigen receptor natural killer) cells are in development, although lifelong immunosuppression via eradication of normal T-cells remains an obstacle to overcome [7].

### 1.2. B-PLL

B-prolymphocytic leukemia (B-PLL) is an extremely rare entity, accounting for ≤1% of all lymphocytic leukemias [35]. Affecting men and women equally often, the median age at diagnosis is 65–69 years [7,35] and prognosis is poor, with a median survival of three years [36].

First described by Galton et al., former diagnostic criteria required ≥55% prolymphocytes in the peripheral blood (characterized by a round nucleus, moderately basophilic cytoplasm and a prominent nucleolus) [37] and mature B-cell-markers detected by immunophenotyping, as well as clonality proven by light chain restriction [6,7]. Clinical presentation typically involves a white blood cell count > 100 × 10^9^/L, splenomegaly, peripheral lymphadenopathy (in 50% of the cases) and B symptoms [7,36].

B-PLL presents with great heterogeneity regarding clinical, biological and molecular features. In 2014, Van der Velden et al. suggested counting B-PLL as a heterogenous subgroup of mantle cell lymphoma (MCL), even in cases negative for t(11;14) [38]. Prolymphocytic transformation may occur in various types of small B-cell lymphomas, and as further proof of their origin, all B-PLL cases show similarities with small B-cell lymphomas [39]. In the latest WHO classification of lymphoid neoplasms, this was taken into account, and B-PLL is no longer recognized as a distinct entity [1]. Cases formerly labeled as B-PLL are now distributed to:

- A blastoid variant of mantle cell lymphoma, characterized by the presence of IGH:: CCND1;
- Prolymphocytic progression of chronic lymphocytic leukemia/small lymphocytic lymphoma (CLL/SLL), defined by CD5-positive non-mantle B-cell neoplasm with >15% prolymphocytes in the peripheral blood and/or bone marrow (cases with <15% of prolymphocytes remain CLL/SLL);
- Splenic B-cell lymphoma/leukemia with prominent nucleoli (comprising former “hairy cell leukemia variant” and specific cases of splenic marginal zone lymphoma as well as CD5-B-PLL cases) [1].

The most frequent genetic changes include abnormalities in TP53 and MYC. However, unlike in other B-cell lymphomas, MYC aberrations in B-PLL cases do not seem to be associated with an aggressive clinical course of the disease [6]. Chapiro et al. suggested discrimination in three risk groups based on cytogenetic findings: low risk (without MYC aberration), intermediate risk (MYC aberration without del17p) and high risk (both MYC aberration and del17p) [40].

This classification determines the choice of the therapy regimen: in absence of high-risk genetic features, immunochemotherapy with a combination of fludarabine, cyclophosphamide, rituximab (FCR) or bendamustin and rituximab (BR) is recommended. For patients with del17p or TP53 mutations, resistance to chemotherapy is likely. Therefore, B-cell receptor pathway (BCR) inhibitors such as ibrutinib are the treatment of choice [7,41]. Eyre et al. reported on the outcome of eight patients receiving the PI3Kδ-inhibitor idelalisib in combination with rituximab, achieving durable remissions (five of eight remained in CR at a median follow-up of 21 months) despite high rates of toxicity [42]. Since treatment strategies for B-PLL are often derived from treatment of CLL, alemtuzumab also constitutes an option in B-PLL [43]. It should be noted that a small subset of patients (10–15%) present with asymptomatic disease, making a “watch and wait” strategy feasible [44].

AlloHSCT might be considered in eligible patients with satisfying responses to initial treatment, as well as in relapse/refractory cases [6,7,41]. Kalaycio et al. reported on 11 cases of B-PLL receiving alloHSCT with a median PFS of 3.5 months [26]. Despite these rather sobering results, case reports describe durable responses after alloHSCT in B-PLL cases [45,46], once more making alloHSCT the only prospect of a cure in selected cases.

### 1.3. Aim of the Present Study

Considering the rarity of both conditions, T-PLL, and particularly B-PLL, and the resulting paucity of outcome data with regard to the curatively intended transplant approach, the aim of this case series is to enhance the available real-world evidence on the outcome of T-PLL and B-PLL patients receiving alloHSCT. Although the size of our study cohort is relatively small (due to the low incidence of the disease [4]), our results provide an important contemporary addition to existing data from retrospective analyses, which were published more than ten years ago [23,24,25,26,27]. Analyzing the data of PLL patients receiving alloHSCT within the last seven years makes our results particularly pertinent, considering the evolution of methods and outcomes of alloHSCT in the past decades.

## 2. Materials and Methods

### Patients and Statistical Methods

This is a retrospective chart analysis of all consecutive alloHSCT performed for T-PLL or B-PLL at our institution between 2015 and 2022.

Acute graft versus host disease (aGvHD) was defined according to the Mount Sinai Acute GVHD International Consortium (MAGIC) criteria [47]. Chronic graft versus host disease (cGvHD) was defined using the National Institutes of Health (NIH) Consensus Development Project on Criteria for Clinical Trials in cGvHD, published in 2015 [48].

All patients gave written informed consent to all transplant procedures and to data collection and analysis, as described in more detail below. Probabilities for overall and progression-free survival were calculated and visualized by the Kaplan–Meier method. Curves were compared by log-rank test. The relapse incidence was calculated by cumulative incidence function considering the competing risk and non-relapse mortality. Statistical analyses and graphics were performed with NCSS 2001 software (NCSS, Kaysville, UT, USA). The Swimmer Plot was created with https://roadmap2health.io/berdapps/swimmer/; accessed on 27 December 2024.

## 3. Results

### 3.1. Baseline Characteristics and Outcomes by T-Versus B-Lineage PLL

Between 1 of January 2015 and 31 of December 2022, nine alloHSCT for patients with PLL were carried out at our institution—six patients suffered from T-PLL, and three patients were diagnosed with B-PLL. According to the novel WHO 2022 classification, two B-PLL cases might be classified as prolymphocytic transformation of CLL, and one case is CD5-negative, and would now be included in the group of splenic B-cell leukemia with prominent nucleoli (SBLP) [1]. Baseline and treatment details, as well as outcome summaries, are listed in Table 3 and Table 4. Below, each case will be individually described.

### 3.2. T-PLL

Between 2015 and 2022, alloHSCT was performed in three male and three female patients diagnosed with T-PLL at our institution. The median age at time of alloHSCT was 64 years (range 52–73, interquartile range (IQR) 58–68.5). Consequently, all patients received RIC regimens, containing lower-dose TBI (2–4 Gy) in three cases. Three patients (50%) received transplantation with a KPS of ≤ 80%. Two patients (33%) were not in remission at the time of transplant.

Regarding transplant characteristics, three patients (50%) received transplantation of a haploidentical family donor, two patients (33%) from an HLA-matched unrelated donor and one patient (17%) had an HLA-identical sibling donor.

Five of six (83%) of the T-PLL patients were in CR at first evaluation via bone marrow biopsy after alloHSCT. Subsequent relapse was documented in four (67%) of our transplanted patients. Three of four (75%) relapsed patients received DLI, and two of three patients (67%) with confirmed relapse achieved CR again and were found to be alive at time of last follow-up. One patient (#5) only had clinically suspected relapse (skin infiltrations and a cerebral mass which could not be histologically examined).

**Figure 1:**
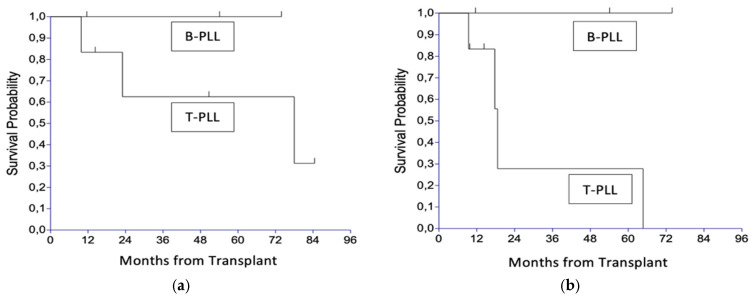
Outcome by lineage. (**a**) Overall survival (OS); (**b**) progression-free survival (PFS).

The median OS of our T-PLL cohort is 78 months (Figure 1a) and the median PFS is 18.6 months (Figure 1b). With a median follow-up of survivors of 50.7 months (range, 14.3–84.5), the 4-year overall survival probability is 62.5% (Figure 1a), with the 4-year PFS probability being 27.8% (Figure 1b).

**Figure 2:**
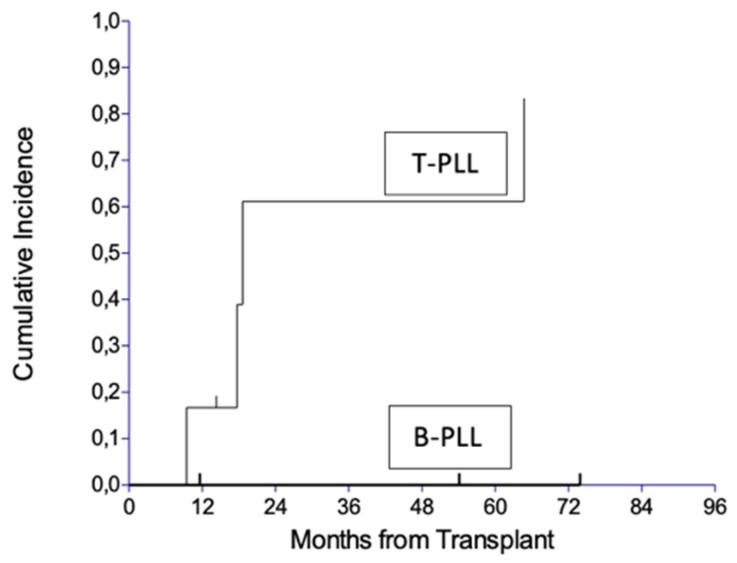
Cumulative incidence of relapse by lineage.

In four of the six patients (67%) with T-PLL relapsing after alloHSCT, the 4-year cumulative relapse incidence in the T-PLL cohort reached 61.1% (Figure 2).

Four out of six patients suffered from aGvHD of at least Grade II, and two patients from cGvHD.

### 3.3. B-PLL

In contrast to T-PLL-patients, two of three patients (67%) received conditioning with myeloablative intensity. None of the B-PLL patients were transplanted in CR1: one in CR2, one in partial remission = PR4 and one patient at the time of his first relapse. At the time of database lock, all three patients are alive without relapse. All three patients suffered from aGvHD, but only one experienced Grade IV lower than GI GvHD. One patient experienced cGvHD.

### 3.4. Impact of Other Clinical Factors on Survival in the Overall Series

The 4-year OS probability of patients in CR at the time of alloHSCT (*n* = 5) was 100%, while that of patients undergoing alloHSCT not in CR (*n* = 4) was 50%. This difference did not reach statistical significance (*p* = 0.13).

**Figure 3:**
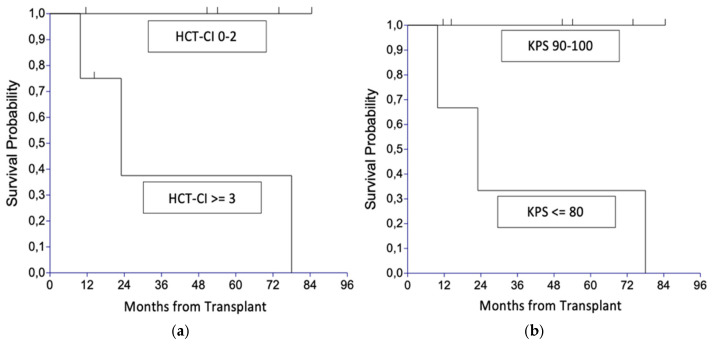
Overall survival (OS) (**a**) by hematopoietic stem cell transplantation comorbidity index (HCT-CI); (**b**) by Karnofsky performance status (KPS).

In contrast to the remission state at alloHSCT, patient-related factors, such as performance score or comorbidity index, had a significant impact on survival, despite the given small size of the presented series. Four-year OS was 100% for patients with a hematopoietic stem cell transplantation comorbidity index (HCT-CI) < 3 at the time of HSCT (*n* = 5), while patients with a pre-transplant HCT-CI of three or higher (*n* = 4) had a 4-year OS probability of only 37.5% (95% confidence interval, 0.0–93.6%; *p* = 0.042); Figure 3a. Similarly, the patients’ pre-transplant KPS had a significant impact on the 4-year OS probability (*p* = 0.028; Figure 3b), with 100% OS for patients with a KPS of 90–100 (*n* = 6), as opposed to a 4-year OS probability of 33% (95% confidence interval, 0.0–86.7%) in patients with KPS of 80 or lower (*n* = 3).

### 3.5. Individual Case Descriptions

**Figure 4:**
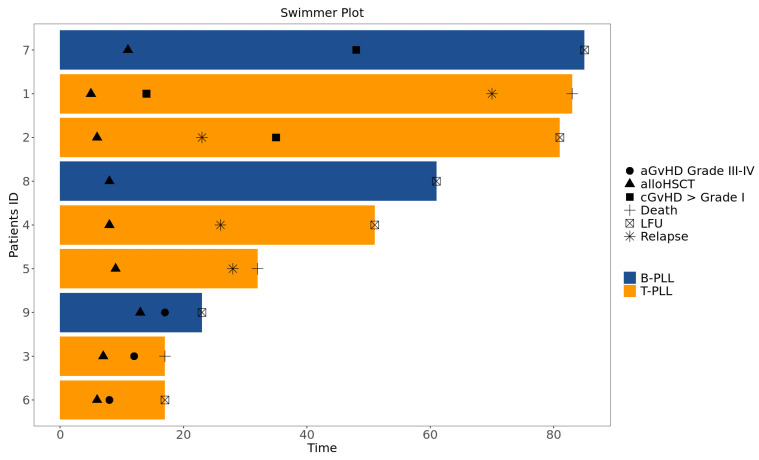
Swimmer plot of individual clinical cases (created with https://roadmap2health.io/berdapps/swimmer/; accessed on 27 December 2024) aGvHD: acute graft versus host disease; cGVHD: chronic graft versus host disease; alloHSCT: allogeneic hematopoietic stem cell transplantation; B-PLL: B-prolymphocytic leukemia; LFU: last follow-up; T-PLL: T-prolymphocytic leukemia.

Before describing every case individually, a swimmer plot visualizing each clinical course is shown in Figure 4.

- Case 1

This patient (female) was diagnosed with T-PLL at the age of 67. Initial characteristics were an excessive WBC count of 400 G/L, as well as a maculopapular rash of the neck and extremities. With CD2, 3, 4, 5 and 7, mandatory surface markers were expressed. TCR was shown to be clonal by PCR, but chromosome analysis was not informative. Alemtuzumab was started, and alloHSCT was carried out in CR1. In addition, aGvHD of the skin and cGvHD with involvement of eyes, skin and liver was diagnosed. Five years and seven months after alloHSCT, a cutaneous relapse occurred, which was treated with a rechallenge of alemtuzumab, DLI and FMC. Despite prolonged cytopenias, the patient initially responded to the chemotherapy regimen, but finally showed progression of the cutaneous infiltrations. Therefore, venetoclax and ruxolitinib were established (venetoclax was approved for CLL at this time, and ruxolitinib was approved for steroid-refractory GvHD since 2022, but was available previously at our center via named patient use) and a satisfying disease control could be achieved. Unfortunately, the patient died of an infectious complication (fungal pneumonia) 77.9 months after alloHSCT.

- Case 2

Patient 2 is a male patient diagnosed with T-PLL at the age of 60. He initially presented with a WBC count of 410 G/L requiring leukapheresis, with splenomegaly of 22 cm and generalized lymphadenopathy. Cytogenetics showed a complex aberrant karyotype. Upon diagnosis, FMC was started, which resulted in CR proven by bone marrow biopsy. AlloHSCT was performed after another course of FMC (dose-reduced to 75% due to neutropenia). AlloHSCT was well tolerated, with no signs of aGvHD. After 1.5 years, relapse occurred. Treatment was initiated with lenalidomide and dexamethasone, and consequently venetoclax due to the persistence of 9% T-PLL cells in the peripheral blood. In addition, a series of four DLI was administered. Thereby, ongoing CR could be achieved, confirmed by bone marrow biopsy.

- Case 3

At the age of 52 years, this female patient was diagnosed with active T-PLL. Notably, one year before, the diagnosis was suspected with a leukocytosis of 25 G/L and a clonal TCR rearrangement, possibly representing an inactive phase of T-PLL, and no treatment was initiated. One year later, the patient presented with upper abdominal pain, which was most likely attributable to lymphadenopathy and splenomegaly (13.5 × 7.5 cm). The diagnosis was established via flow cytometry (CD52+) and bone marrow biopsy, which showed 80% infiltration. Cytogenetics revealed a regular female karyotype. Alemtuzumab was initiated as the first line treatment. Unfortunately, after 4 months, the patient presented with clinical progress manifesting with effusions as well as skin infiltration. Salvage therapy with FMC as well as intrathecal prophylaxis with methotrexate (MTX), cytarabine and dexamethasone was established. Due to further progress, salvage therapy was switched to venetoclax, again without response, and alloHSCT was carried out with refractory disease. Because of residual T-PLL cells in the bone marrow (7% in the bone marrow biopsy), the patient received lenalidomide. Thereby, the patient achieved CR. Nevertheless, recurrent paracentesis due to pleural effusions and ascites was necessary—although not diagnostically proven, hepatic cirrhosis was suspected in this patient. She died 9.8 months after alloHSCT without evidence for relapsed T-PLL.

- Case 4

This male patient was diagnosed with T-PLL at the age of 72. Leukocytosis of 30 G/L was present, and 80% of the leukocytes were T-PLL type. TCR was clonal, and cytogenetics were partially informative, suggesting a loss of Y chromosome in six representative metaphases. After one course of FMC, alemtuzumab was administered and haploidentical HSCT could be carried out in CR1. After 19 months, relapse occurred. After one cycle of FMC followed by DLI, CR could be reestablished. Mild and transient cutaneous GVHD occurred, and CR of T-PLL is currently ongoing.

- Case 5

This 68-year-old woman was diagnosed with T-PLL with only mild leukocytosis of 15 G/L, but 70% bone marrow infiltration. Cytogenetics showed a complex karyotype; characteristic 14q11 rearrangement was found as well. The patient received alemtuzumab and underwent alloHSCT in CR1 (haploidentical bone marrow transplant), following which she could achieve CR. Unfortunately, this patient was diagnosed with EBV-associated post-transplant lymphoproliferative disorder (PTLD) 6 months after alloHSCT, which was treated with repeated doses of rituximab. Thereby, disease control could be achieved. Subsequently, the patient presented with erythroderma with skin infiltrations initially suspected to represent GvHD. However, repeated biopsies could not confirm GvHD, although CD3+ T-cell infiltrates were shown, and high-dose corticosteroids were necessary to control the severe erythroderma. When the patient further presented with cerebral lesions, biopsy was not feasible, and due to rapid clinical deterioration, best supportive care measures were established. We clinically suspect a cutaneous and central-nervous relapse of T-PLL, taking into consideration that EBV (blood and liquor) could not be detected any more. Further, the clinical presentation and steroid refractoriness of the cutaneous T-cell infiltrates were not suggestive for GvHD. The patient died one year and eleven months after alloHSCT following clinically suspected relapse of the original disease.

- Case 6

The diagnosis of T-PLL in this 60-year-old male patient was made by pathological analysis of cervical lymph nodes; bone marrow showed 50% infiltration. There was no leukocytosis evident, but despite the cervical and mesenterial lymphadenopathy, a mild splenomegaly (12 × 4.8 cm) and skin infiltrations (initially interpreted as mycosis fungoides) were present. Cytogenetics showed a complex aberrant karyotype. Alemtuzumab induced CR, and alloHSCT was carried out. Despite aGvHD of the skin and upper and lower GI tract, no further complications occurred, and the patient remains in CR.

- Case 7

In this male patient, B-CLL Binet A was diagnosed 13 years in advance to prolymphocytic transformation. Transformation to Binet stage B occurred 2 years after diagnosis. Three courses of FCR were administered 3 years later, followed by another three courses due to progression. One year prior to transplant, 13% CD5+ lymphocytes with prolymphocyte transformation were detected in the peripheral blood via flow cytometry; aberrant cytogenetics were complex. Histological examination of a lymph node revealed infiltration of CD5+ cells with prolymphocytic transformation. Bone marrow biopsy showed 10–15% infiltration of CD5+ lymphocytes. Therefore, the disease would have been classified as prolymphocytic transformation of CLL according to the WHO 2022 classification [1]. The patient received seven courses of rituximab and idelalisib followed by obinutuzumab-CHOP, resulting in partial remission. Hereupon, alloHSCT was performed, through which the patient achieved ongoing CR.

- Case 8

When the diagnosis of B-CLL/B-PLL was made, this male patient was 69 years old. With 15% CD5+ lymphocytes in the peripheral blood, according to the WHO 2022 classification, this case represents prolymphocytic transformation of CLL [1]. Nevertheless, NOTCH1 mutation was detected. Mutations in the NOTCH pathway are related to marginal zone lymphoma [39], and NOTCH2 mutation can be found in up to 25% of patients with marginal zone lymphoma [41]. Therefore, it could be argued that the case counts as splenic B-cell leukemia with prominent nucleoli, as described in WHO 2022 [1]. Cytogenetic alterations included the loss of TP53, which in the absence of MYC aberrations, does not contribute to the high-risk group of B-PLL cases [39]. The patient received two courses of immunochemotherapy with fludarabine/epirubicin/rituximab and consecutively alemtuzumab. Despite initially achieving molecular CR confirmed by bone marrow examination, in the pre-transplant re-evaluation via bone marrow biopsy, 70% lymphocytes were found cytologically. Transplant was thus carried out for relapsed disease. Despite aGvHD of the upper GI and skin, no further complications occurred, and the patient remained in CR until last follow-up.

- Case 9

This male patient was diagnosed with B-PLL at the age of 63. He presented with splenomegaly (24 cm) and elevated LDH levels. Notably, CD5 was not expressed on the prolymphocytes. In the pathological workup, the cells were described as round cells with prominent nucleoli, typical for B-prolymphocytes [37]. Cytogenetics showed loss of chromosome Y and del(4p16), and via fluorescence in situ hybridization (FISH), a subclone with MYC aberration was evident. As the group of “splenic B-cell leukemia with prominent nucleoli” also includes CD5-negative B-PLL cases, this would retrospectively be the appropriate group of B-PLL according to the latest WHO classification [1]. The patient received ibrutinib for 5 months, but unfortunately experienced progression while on treatment. Via obinutuzumab–bendamustin, CR was achieved and alloHSCT with an HLA-identical sibling donor was carried out consecutively. The patient developed severe, steroid refractory gastrointestinal GvHD, eventually responding after multiple lines of immune interventions.

## 4. Discussion

Unfortunately, virtually all T-PLL patients will relapse eventually after a median response duration of 12 months [16,19,20]. Used as a consolidation therapy, alloHSCT offers the only prospect of a cure for patients with T-PLL [11]. Yet, only 30–50% of the patients are eligible for this intensive therapy [15].

To achieve profound disease control either in first or second remission, studies suggest alloHSCT should be evaluated for all patients [4,15]. 3-year OS rates of approx. 34% have been reported [23,25,27]. The main challenges are non-relapse mortality (NRM) and relapse after alloHSCT; the latter is associated with particularly dismal outcome [6].

With a median age of 64 years in our cohort, T-PLL patients were older than in previous transplant studies, in which the median age was <57 years [23,24,25,26,27]. In addition, with 50%, the rate of a KPS ≤ 80% was slightly higher compared to other studies (Kalacyio et al. and Wiktor-Jedrzejczak et al. had a rate of 25% and 31%, respectively [26,27]).

Despite the high rates of CR1 (80%) at the time of transplantation, relapse occurred in four out of six (67%) of our T-PLL patients, which is higher compared to previous studies. Krishnan et al. and Wiktor-Jedrzejczak et al. reported relapse rates <40% [23,27]. Two of the relapsed cases (50%) (#2, #5) had a complex aberrant karyotype at diagnosis. This might have contributed to early relapse, considering complex karyotype as an established risk factor for relapse [14]. Furthermore, three of the four (75%) relapsed patients were older than 62 years at time of diagnosis, which is also associated with worse prognosis [17]. Interestingly, three of the relapsed patients received salvage chemotherapy and DLI and could thereby achieve CR again, two of them ongoing, while the third patient succumbed to infection while leukemic skin infiltrates were responding to therapy.

Due to the prompt and successful relapse management in two of the four relapsed patients, the overall outcome of our T-PLL cohort appears favorable in comparison with previous studies, with a median OS of 78 months, while Wiktor-Jedrzejczak et al. described an OS of 27.8 months [27], and Krishnan et al. reported a median OS of 33 months [23]. The median PFS in our T-PLL series of 18.6 months is similar to that reported by Wiktor-Jedrzejczak et al. (19.2 months) [27], while Kalaycio et al. described an even shorter median PFS of 5 months for the T-PLL patients receiving alloHSCT [26]. Importantly, our favorable outcome data refers to a small study cohort of only nine patients.

The significance of the appropriate timing of donor lymphocyte infusions (DLI) becomes evident as the effectiveness of the graft-versus-leukemia (GvL) effect appears to be uncertain in T-PLL patients [24], even more than in other lymphoid malignancies [27]. The GvL effect can be shown via reduction in minimal residual disease (MRD) but is often temporary in T-PLL patients [7]. Thus, MRD monitoring might be useful to select appropriate timing for donor lymphocyte infusion (DLI) [16].

In our T-PLL cohort, the median time from diagnosis to transplant was only 6 months, which may have contributed to the relatively favorable outcome, since Wiktor-Jedrzejczak et al. found that a short time span between diagnosis and alloHSCT (<12 months) is associated with better outcome rates [24]. However, the first prospective study regarding alloHSCT in T-PLL patients, published in 2019 by Jedrzecjak et al., could not show association between a short time interval between diagnosis and alloHSCT and even increased NRM rates occurred [27]. Yet, risk of relapse was again significantly reduced by performance of TBI ≥ 6 Gy as part of the conditioning regimen.

Furthermore, all previous retrospective studies on alloHSCT in T-PLL patients, as summarized in Table 1 and Table 2, were published before 2015 [23,24,25,26]. Importantly, supportive care measures and general alloHSCT management improved over the past years, which makes superior outcomes feasible.

All of our six T-PLL patients received RIC, while in the analyses by Krishnan et al., Wiktor Jedrzejczak et al. in 2012 and Kalaycio et al. [23,24,26], only approx. 30% of the patients underwent RIC. This may have contributed to the relatively low rate of transplant-related mortality in our cohort.

The outcome of our small cohort of patients receiving alloHSCT for B-PLL was excellent; all were alive and relapse-free, with a post-transplant follow-up of 74, 54 and 12 months, respectively.

Kalaycio et al. described a median post-alloHSCT PFS of 3.5 months for B-PLL, which is considerably shorter than in our cohort [26]. Comparison with available data appears challenging due to the heterogeneity of B-PLL cases regarding morphological features, as well as the clinical course [39], ranging from indolent to aggressive development [41]. However, according to the risk stratification by Chapiro et al., one patient (#9) had an intermediate risk (MYC aberration in the absence of del17p) while none of the patients presented with both MYC aberration and del17p, which would have been considered the highest risk category by Chapiro et al. [40]. Nevertheless, our two B-PLL cases without MYC aberration had either loss of TP53 (case #8) or a complex aberrant karyotype (case #7), and both had failed one or more previous treatment lines.

With three out of nine (33%) alloHSCTs carried out with haploidentical donors, our series is not powered to conduct conclusions about the impact of donor type on the outcome of our PLL patients. However, the use of post-transplant cyclophosphamide (PTCy)-based immunosuppression, which is particularly effective in preventing severe cGvHD [49], may be another important cause for the favorable outcomes of the presented series. In addition, none of our patients was transplanted with a mismatch unrelated donor. Furthermore, all HLA-matched transplants, including related and unrelated alloHSCT, have applied algorithms for the use and dosage of anti T-lymphocyte globulin (ATLG), considering established GVHD risk factors such as recipient–donor relationship and sex mismatch, but also emerging risk factors as the HLA-C killer cell immunoglobulin-like receptor (KIR) ligand status [50], the pre-ATLG lymphocyte count [51] and adaption of the ATLG dose to the post-grafting use of MTX versus mycophenolate mofetil (MMF) [52].

Regarding the role of alloHSCT in PLL in the era of modern T-cell-engaging immunotherapies, novel treatments such as bispecific antibodies and CAR-T-cells may serve as bridging to alloHSCT (similar to their role in B-cell acute lymphoblastic leukemia [53,54]), given their high response rates, even in otherwise refractory disease [7].

## 5. Conclusions

In conclusion, the favorable outcomes of our case series of PLL patients receiving alloHSCT confirm the potential of alloHSCT to provide the chance for long-term disease control in this heterogeneous group of malignancies with otherwise extremely poor prognoses. Notably, this also seems to be the case for B-PLL, for which particularly poor alloHSCT outcomes have previously been reported. The small patient number and retrospective nature of our study demands caution with the interpretation of data. Nevertheless, we can draw some interesting conclusions. One important finding from our small series is the fact, that in two cases of T-PLL post-alloHSCT relapse could be effectively managed with merely short courses of salvage (chemo-)therapy followed by DLI, which lead to the restoration of sustained CR in both cases. These T-PLL cases, as well as the excellent disease control in our small B-PLL series, are highly suggestive of a significant GvL effect being operative after alloHSCT in this group of diseases. Although disease control prior to alloHSCT may be as important a prognostic factor for post-alloHSCT outcomes in PLL as in other diseases, our series was not sufficiently powered to confirm this assumption. On the other hand, despite the small size of our unicentric case series, a significant effect on survival of the patient-related factors, i.e., the general condition assessed by the KPS, and comorbidities expressed by the HCT-CI, could be detected. The assessment of transplant feasibility in the given, elderly patient population should, thus, primarily take into account these important patient-related factors.

## Figures and Tables

**Table 1:** Comparison of patient and transplant characteristics in previous studies.

Author	Study Design	No. of Patients with T-PLL Receiving alloHSCT	Median Age at Time of alloHSCT (Range)	Karnofsky Index at Time of alloHSCT >80% (% of Patients)	Median Time in Months from Diagnosis to alloHSCT (IQR)	Remission Status CR at Time of alloHSCT (% of Patients)	Remission Status PR at Time of alloHSCT (% of Patients)	Remission Status Refractory (resp. Stable) at Time of alloHSCT (% of Patients)
Krishnan et al., *British Journal* *Of Haematology*, 2010 [23]	Multicenter retrospective analysis	13	51 (39–61)	n.a.	n.a.	9 (69)	4 (31)	0
Kalaycio et al., *Biology of Blood Marrow Transplantation*, 2010 [26]	Retrospective analysis of the CIBMTR database	21	54 (30–75) ^1^	30 (75) ^1^	11 (2–78) ^1^	16 (36)^1^	8 (18) ^1^	21 (46) ^1^
Wiktor-Jedrzejczak et al., *Leukemia*, 2012 [24]	Retrospective analysis of the EBMT database and the Royal Marsden Consortium	41	51 (24–71)	n.a.	12 (4–58)	11 (27)	12 (29)	13 (32)
Guillaume et al., *European Journal of Hematology*, 2014 [25]	Retrospective analysis of the SFGM-TC database	27	53 (36–65)	n.a.	8.5 (4.5–59)	14 (52)	10 (37)	3 (11)
Wiktor- Jedrzejczak et al., *Bone Marrow Transplant*, 2019 [27]	**Prospective** observational study based on the EBMT registry	37	56(47–59) ^2^	24 (69)	8 (6–17)	16 (44)	8 (22)	4 (11)

Abbreviations: alloHSCT: allogeneic hematopoietic stem cell transplantation; CIBMTR: Center for International Blood and Marrow Transplant Research; CR: complete remission; EBMT: European Group for Blood and Marrow Transplantation; IQR: interquartile range; n.a.: not available; PR: partial remission; SGFM-TC: Registry in French Society for Stem Cell Transplantation. ^1^ concerning the whole cohort (47 patients) incl. B-PLL and PLL not further specified, ^2^ only interquartile range (IQR) available.

**Table 2:** Summary of transplant characteristics and outcomes in previous studies.

Author	RIC (%)	Median OS in Months	Median PFS in Months	3-Year OS (95% CI)	3-Year PFS (95% CI)	Cumulative Incidence of Relapse	Cumulative Incidence of NRM
Krishnan et al., *British Journal* *Of Haematology*, 2010 [23]	4 (31)	33	n.a.	38%	38%	31%	31%
Kalaycio et al., *Biology of Blood Marrow Transplantation*, 2010 [26]	14 (30) ^1^	n.a.	5	n.a.	n.a.	39% within one year ^1^	28% within one year ^1^
Wiktor-Jedrzejczak et al., Leukemia, 2012 [24]	13 (31)	12	10	21%	19%	41% within 3 years	41% within 3 years
Guillaume et al., *European Journal of Hematology*, 2014 [25]	16 (59)	n.a.	n.a.	36% (17–54)	26% (14–45)	47% within 3 years	31% within 3 years
Wiktor-Jedrzejczak et al., *Bone Marrow Transplant*, 2019 [27]	24 (65)	27.8	19.2	42% (25–59) ^2^	30% (14–46) ^2^	38% within 4 years	32% within 4 years

Abbreviations: CI: confidence interval; n.a.: not available; NRM: non-relapse mortality; OS: overall survival; PFS: progression-free survival; RIC: reduced intensity conditioning ^1^ concerning the whole cohort (47 patients) incl. B-PLL and PLL not further specified ^2^ 4-year OS and 4-year PFS.

**Table 3:** Baseline and transplant characteristics and outcome summary of six T-PLL patients receiving alloHSCT at our institution.

Case	Sex	Age at Time of allo HSCT	Cytogenetics	Donor type	Remission Status at Time of alloHSCT	HCT-CI	Karnofsky Index	Conditioning Details	GvHD Prophylaxis	aGvHD II	aGvHD III-IV	cGvHD II-III	Relapse	DLI	OS in Months	PFS in Months	LFU
1	f	67	n.a.	MUD	CR1	3	80	FB2-ATLG25 (RIC)	CSA-MMF	Skin	N	Oral, Eyes, Liver	Y	Y	78	64.7	D
2	m	61	Complex aberrant karyotype (legend “T2”)	MUD	CR1	0	90	FB2-ATLG40 (RIC)	CSA-MMF	N	N	Oral, Eyes, Liver, Skin	Y	Y	76	17.7	A
3	f	52	Regular	MSD	Refractory	3	50	FC-VP16-TBI4Gy (RIC)	CSA-MMF		Skin, Upper + Lower GI	N	N	N	9.8	9.8	D
4	m	73	Loss of chromosome Y	Haplo	CR1	2	90	Cy30-FB2 (RIC)	PTCy-TAC-MMF	N	N	N	Y	Y	43.8	18.6	A
5	f	68	Complex aberrant karyotype (legend “T5”)	Haplo	Refractory	3	80	Thio4-Flu-TBI2Gy (RIC)	PTCy-TAC-MMF	Upper GI	N	N	Y	N	23	19	D
6	m	61	Complex aberrant karyotype (legend “T6”)	Haplo	CR1	4	90	Thio4-Flu-TBI2Gy (RIC)	PTCy-TAC-MMF		Lower GI	N	N	N	12	12	A

A: alive; aGvHD: acute graft versus host disease; alloHSCT: allogeneic hematopoietic stem cell transplantation; ATLG: anti-T-lymphocyte globulin; B: busulfan; Cy: cyclophosphamide; CR: complete remission; CSA: cyclosporine A; D: dead; DLI: donor lymphocyte infusion; F: fludarabine; GI: gastrointestinal; GvHD: graft versus host disease; Haplo: haploidentical donor; HCT-CI: hematopoietic cell transplantation specific comorbidity index; LFU: last follow up; MMF: mycophenolate mofetil; MSD: matched sibling donor; MUD: matched unrelated donor; N: none; n.a.: not available; OS: overall survival; PFS: progression free; PTCy: post-transplant cyclophosphamide; RIC: reduced intensity conditioning; TAC: tacrolimus; TBI: total body irradiation; Thio: thiotepa; VP16: etoposide; Y: yes. Karyotype details: T2: 45,XY, der(4)(del)(q11)t(4;11),+8,i (8) x2, der(11)t(4;11)(q11;p12), der (13;21)(q10;q10), inv(14)(q11q32), der (15)t(15;22)dup(22), -22; T5: 45~46,XX,?add(12)(q24),-14,-17,+3mar; T6: 43,X,-Y,add(5)(p15),i(8)(q10),del(11)(q22),add(12)(q24),-12,-14,+21,idic(21)(p12),add(22)(q13).

**Table 4:** Baseline and transplant characteristics and outcome summary of three B-PLL patients receiving alloHSCT at our institution.

Case	Sex	Age at Time of alloHSCT	Cytogenetics	Donor Type	Remission Status at Time of alloHSCT	HCT-CI	Karnofsky Index	Conditioning Details	GvHD Prophylaxis	aGvHD II-IV	aGvHD III-IV	cGvHD II-III	Relapse	DLI	OS	PFS	LFU
7	m	55	Complex aberrant karyotype (legend “B7”)	MSD	PR4	0	90	FB2-TBI4Gy (MAC)	CSA-MTX	GI, Skin	N	Oral	N	N	73.9	73.9	A
8	m	69	Loss of TP53	MUD	Relapse 1	0	90	FB2-ATLG45-TBI4Gy (MAC)	CSA-MMF	Upper GI + Skin	N	N	N	N	54.1	54.1	A
9	m	64	Loss of chromosome Y and del4p16	MSD	CR2	2	100	Thio8/Flu/TBI2GyATLG25 (RIC)	CSA-MMF		Lower GI	N	N	N	11.1	11.1	A

A: alive; aGvHD: acute graft versus host disease; alloHSCT: allogeneic hematopoietic stem cell transplantation; ATLG: anti-T-lymphocyte globulin; B: busufan; cGvHD: chronic graft versus host disease; CR: complete remission; CSA: cyclosporine A; DLI: donor lymphocyte infusion; F: fludarabine; GI: gastrointestinal; GvHD: graft versus host disease; HCT-CI: hematopoietic cell transplantation-specific comorbidity index; LFU: last follow-up; MMF: mycophenolate mofetil; MSD: matched sibling donor; MUD: matched unrelated donor; N: none; OS: overall survival; PFS: progression-free; PR: partial remission; RIC: reduced intensity conditioning; TBI: total body irradiation; Thio: thiotepa. Karyotype details: B7: three pathologic subclones were identified: 45,X,t(Y;8)(q10;q10); 46,XY,t(2;7)(p11;q32),t(5;9)(p15;q22); 46,XY,-6,+mar.

## Data Availability

Data are available from the authors upon request.

## Abbreviations

alloHSCT	Allogeneic hematopoietic stem cell transplantation
ATLG	Anti-T-lymphocyte globulin
ATM	Ataxia teleangiectasia mutated
aGvHD	Acute graft-versus-host disease
autoHSCT	Autologous hematopoietic stem cell transplantation
BCL	B-cell lymphoma
BCL-XL	B-cell lymphoma extra-long
B-PLL	B-prolymphocytic leukemia
BCR	B-cell receptor
B2M	Beta-2-microglobulin
CAR-NK	Chimeric antigen receptor natural killer
CDK9	Cycline-dependent kinase 9
cGvHD	Chronic graft-versus-host disease
CHOP	Cyclophosphamide, doxorubicin, vincristine, prednisone
CI	Confidence interval
CIBMTR	Center for International Blood and Marrow Transplant Research
CLL	Chronic lymphocytic leukemia
CNS	Central nervous system
CR	Complete remission
DLI	Donor lymphocyte infusion
DNA	Deoxyribonucleic acid
EBMT	European Group for Blood and Marrow Transplantation
ECP	Extracorporeal photopheresis
EZH2	Enhancer of zeste homolog 2
FCR	Fludarabine, cyclophosphamide, rituximab
FISH	Fluorescence in situ hybridization
FMC	Fludarabine, mitoxantrone and cyclophosphamide
GvL	Graft-versus-leukemia
HCT-CI	Hematopoietic stem cell transplantation comorbidity index
HDAC	Histone deacetylase
HTLV	Human T-lymphotropic virus
IL2RG	Interleukin 2 receptor gamma
IQR	Interquartile range
JAK/STAT	Janus-activated kinase signal transducer and activator of transcription factor
KPS	Karnofsky performance status
LDH	Lactate dehydrogenase
LFU	Last follow-up
MCL	Mantle cell lymphoma
MCL-1	Myeloid cell leukemia 1
MMF	Mycophenolate mofetil
MAGIC	Mount Sinai Acute GVHD International Consortium
MRD	Minimal residual disease
MSD	Matched sibling donor
MTCP1	Mature T-cell proliferation 1
MTX	Methotrexate
MUD	Matched unrelated donor
MYC	Master regulator of cell cycle entry and proliferative metabolism
n.a.	Not available
NIH	National Institutes of Health
NK	Natural killer
NRM	Non-relapse mortality
ORR	Overall response rates
OS	Overall survival
PARP	Poly-ADP-ribose polymerase
PCR	Polymerase chain reaction
PFS	Progression-free survival
PI3K	Phosphoinositide 3-kinase
PR	Partial remission
PTCy	Post-transplant cyclophosphamide
PTLD	Post-transplant lymphoproliferative disorder
R/R	Relapsed/refractory
RIC	Reduced intensity conditioning
RTC	Reduced toxicity conditioning
SBPL	Splenic B-cell leukemia with prominent nucleoli
SGFM-TC	Registry in French Society for Stem Cell Transplantation
SLL	Small lymphocytic lymphoma
TBI	Total body irradiation
TCL1	T-cell leukemia/lymphoma 1
TCR	T-cell receptor
T-PLL	T-prolymphocytic leukemia
TRM	Transplant-related mortality
WBC	White blood cell
WHO	World Health Organization
